# Are radiographic indices reliable indicators for quantitative bone mineral density and vitamin D status after femoral neck fractures? A retrospective study in 112 elderly patients

**DOI:** 10.1186/s13037-015-0085-2

**Published:** 2015-11-25

**Authors:** Andy K. S. Yeo, Annette B Ahrberg, Jan D. Theopold, Sebastian Ewens, Gudrun Borte, Christoph Josten, Johannes K. M. Fakler

**Affiliations:** Department of Orthopaedic Surgery, Changi General Hospital, 2 Simei Street 3, Singapore, 529889 Singapore; Department of Orthopaedic, Trauma and Plastic Surgery, University of Leipzig, Liebigstr. 20, 04103 Leipzig, Germany; Department of Diagnostic and Interventional Radiology, University of Leipzig, Liebigstr. 20, 04103 Leipzig, Germany

**Keywords:** Bone architecture, Cortical thickness, Femoral geometry, Bone mineral density, Osteoporosis, Vitamin D deficiency, Femoral neck fracture, Radiographs

## Abstract

**Background:**

Radiographic parameters and indices obtained from hip x-rays are a potential tool to promptly estimate bone quality in elderly hip fracture patients. Preoperative decision in whether to use cemented or cement augmented implants might be supported by this information and thus improve patient safety. Subsequently, this study was conducted to evaluate radiographic parameters as a prescreening tool for bone quality.

**Methods:**

A retrospective analysis of 112 elderly patients with a femoral neck fracture after low-energy trauma was performed (81 % female, 19 % male). Three radiological indices were calculated on hip x-rays: cortical index antero-posterior CTI (ap), cortical index lateral CTI (lat) and canal to calcar ratio CCR. These indices were analyzed for correlations with DXA T-Scores and serum 25-hydroxyvitamin D (25(OH)D) using the Spearman test.

**Results:**

Median age of patients was 80 (IQR 72–86) years. A linear correlation was found for CTI (lat) and T-Score at the total hip (*p* < 0.001, *r* = 0.589), femoral neck (*p* = 0.005, *r* = 0.405) and the lumbar spine (*p* = 0.002, *r* = 0.299). A significant correlation was also indicated between CTI (lat) and 25(OH)D (*p* = 0.002, *r* = 0.293). CTI (lat) at a cut-off level of 0.4 showed a sensitivity of 79 % and a specificity of 56 % in predicting a T-score ≤ −2.5 at the total hip. Gender specific analysis revealed a higher sensitivity (100 %) and specificity (73 %) of CTI (lat) at a cut-off level of 0.4 for men. For severe vitamin D deficiency (<10 ng/ml) sensitivity and specificity were 75 % and 65 %.

**Conclusion:**

Radiographic indices as the CTI (lat) exhibit a direct correlation to BMD and serum 25OH vitamin D levels. A CTI (lat) cut-off level of 0.4 is recommended for identifying patients at risk of osteoporosis expressed by T-Scores ≤ −2.5 and severe vitamin D deficiency.

## Introduction

Osteoporosis has been a growing problem for the past few years. With the increase in the proportion of geriatric patients within the population, the scale of the problem has compounded. Increasingly, hip fracture has become not just a clinical problem with high mortality but also a problem of healthcare costs [1]. Multiple countries have pathways and systems in place to ensure the expedient treatment and care of patients with hip fractures in efforts to minimise mortality as well as in hospital stay [2].

Apart from an increased risk of further fractures, osteoporosis impedes stable fixation of implants putting osteosynthetic devices at high risk for failure despite correct reduction and implant position [3, 4]. Reduced bone mineral density (BMD) due to low mineralisation of the bone is one aspect in compromised bone quality found in osteoporosis. BMD can be measured by dual-energy X-ray absorptiometry (DXA) which still is the gold standard in diagnosing and monitoring osteoporosis. However, BMD is not without its limitations. BMD is a measure of the aggregate density of a certain area. The averaging of density does not take into account the relative sizes of the cortical versus the cancellous component of the femoral neck [5–7]. Another diagnostic tool in classifying bone quality is quantitative computed tomography (QCT) which is based on the principle of volumetric analysis of the bone structure rather than an areal measurement the DXA scan performs. By estimating the cortical bone and cancellous bone in a 3-dimensional model, it can more accurately approximate the amount of bone present. The disadvantage is the cost and availability, as well as the much higher radiation exposure. Also, there are no current comparative studies with DEXA as a gold standard or clinical correlational studies. The advantage may be only in isolated situations and study purposes, but are not recommended for screening [8, 9].

Preoperative DXA as well as QCT are usually not available in the acute setting of hip fracture and urgent surgery. Consequently, these tools do not support the orthopaedic surgeon in deciding whether to use cement augmentation or what type of implant to choose. Lately, mechanical torque-testing devices were developed for intraoperative measurement of bone strength [10]. This device showed a good correlation with BMD in cadaveric femurs [11], but clinical experience with this device is still limited [12].

Alternatively, radiographs of the hip are readily available at low costs. Characteristic radiomorphological aspects as the thinning of the trabecular pattern in the proximal femur described by the Singh index can identify abnormal bone loss and consecutively a higher risk of osteoporosis [13]. But results regarding the accuracy of the Singh index in estimating osteoporosis are conflicting [14, 15]. Another method to classify bone quality with radiographs of the hip was introduced by Dorr and colleagues. They identified radiographic parameters and indices that correlated with bone quality validated by histologic examinations [16]. Furthermore, some of these radiographic parameters demonstrated a significant correlation with BMD measured by DXA in female patients with coxarthrosis scheduled for total hip arthroplasty (THA) [17].

Apart from BMD, low levels of 25-hydroxyvitamin D (25(OH)D) were suggested to act as an additional important factor in increased fragility of the femoral neck and consequently bone health [18]. Since orthogeriatric patients demonstrate a high prevalence of severe vitamin D deficiency [19, 20], a relationship between serum 25(OH)D levels and radiographic parameters was hypothesized.

We designed this study to evaluate the applicability of the radiographic parameters and indices described by Dorr et al. [16] for estimation of bone quality in elderly patients with an osteoporotic fracture of the femoral neck following low-energy trauma.

## Patients and methods

This retrospective analysis included charts and x-rays from 112 patients with a femoral neck fracture, treated at the senior author’s institution between 2011 and 2014. Female patients counted for 81 % (*n* = 91), male patients for 19 % (*n* = 21). Inclusion criteria was age over 50 years for female and over 60 years for male patients who sustained a medial femoral neck fracture after low energy trauma. Exclusion criteria included patients who had a high energy trauma or pathological fractures. Written informed consent was obtained from all patients or their legal guardian.

BMD was measured by DXA (Hologic Delphi A (S/N71109), Bedford, USA) within 10 days after surgery in all patients. 104 (93 %) patients received DXA of the spine, 52 (46 %) of the hip. Osteoporosis was considered at T-Scores at or below −2.5 [5–7]. 48 % (*n* = 54) of these patients with low-energy-trauma and subsequently assumable osteoporotic fracture presented a T-Score ≤ −2.5.

**Fig. 1:**
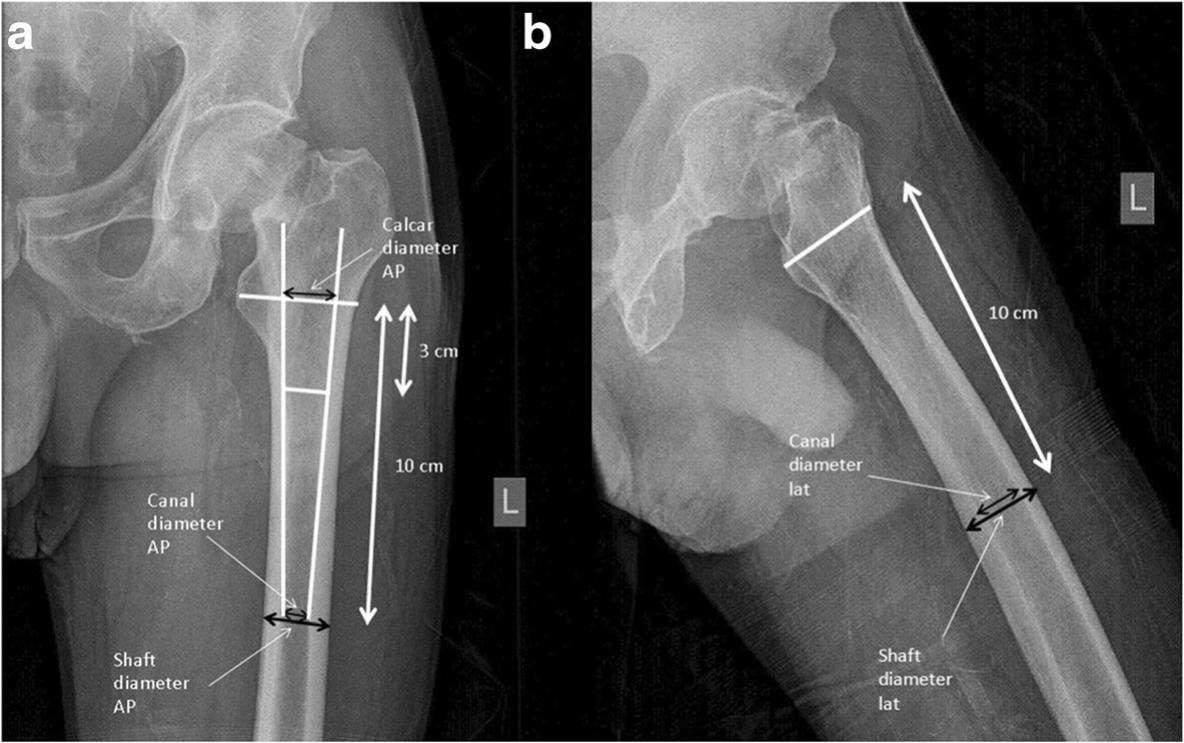
Antero-posterior view (**a**) and lateral (**b**) radiographs of the hip demonstrating the area of radiographic measurements

The radiographs were reviewed and radiographic parameters measured by an orthopaedic surgeon. Anteriorposterior (AP) and lateral Lauenstein (LAT) hip radiographs were obtained preoperatively and on the first or second postoperative day. If preoperative x-rays were not adequate for radiomorphometric analysis (38 %), postoperative x-rays were used for analysis. For instance, this was necessary in case of considerable external rotation of the femoral shaft due to the medial femoral neck fracture seen on some of the AP views. Postoperative AP x-rays were obtained with the affected lower extremity in a standardized position (10° internal rotation of the affected leg and patella cantered in the midline and apex of the knee). If measurements could not be established in pre- or postoperative x-rays, patients were excluded. Radiographic data were measured on AP and LAT view x-rays (Fig. 1) as described by Dorr et al. [16]. Cortical thickness was measured 10 cm distal and parallel to the mid-lesser trochanter line in AP as well as LAT views [Fig. 1]. Femoral diaphysis width (DW) minus medullary canal width (FW) divided by DW resulted in the CTI for each radiographic view [16, 17]. Intrarater reliability in measuring CTI in both AP and LAT views was demonstrated to be 96 % and 94 % [17]. Radiographic measurements were performed on digital x-rays using implemented MagicWeb® software (Visage Imaging GmbH, Berlin, Germany).

Blood samples were taken on admission. Routine blood tests were analysed by commercially available assays. Serum 25(OH)D was determined with a LIAISON® 25-OH Vitamin D assay (DiaSorin, Stillwater, MN, USA). 25(OH)D levels below 10 ng/ml were defined as severe vitamin D deficiency [21].

Statistical analysis was performed with SPSS Version 20 (IBM, Armonk, USA). Values are given as median and interquartile range (IQR, 25.-75. percentile). Except age, all other variables did not show a normal distribution. Consequently, non-parametric methods as the Mann–Whitney-*U* test and the Spearman test were performed. Categorical variables are shown as proportions and the differences between the groups were analyzed using the *χ*2 – test. *p* < 0.05 was considered as statistically significant.

## Results

Median age of 112 included patients was 80 (IQR 72–86) years. Median T-Scores for spine, total hip and femoral neck were −1.5 (IQR −2.7 to −0.7), −2.5 (IQR −3.1 to −2.1) and −3.0 (IQR −3.9 to −2.3). Lumbar and total hip T-Score (*p* < 0.001, *r* = 0.630), lumbar and femoral neck T-Score (*p* < 0.001, *r* = 0.652) as well as total hip and femoral neck T-Score (*p* < 0.001, *r* = 0.798) exhibited a strong correlation. Overall only 48 % presented with T-Score values ≤ −2.5. Lumbar spine T-Scores ≤ −2.5 were detected in only 31 % of the examinations. At the total hip and femoral neck T-Scores indicating osteoporosis were found in 55 % and 71 %, respectively.

**Fig. 2:**
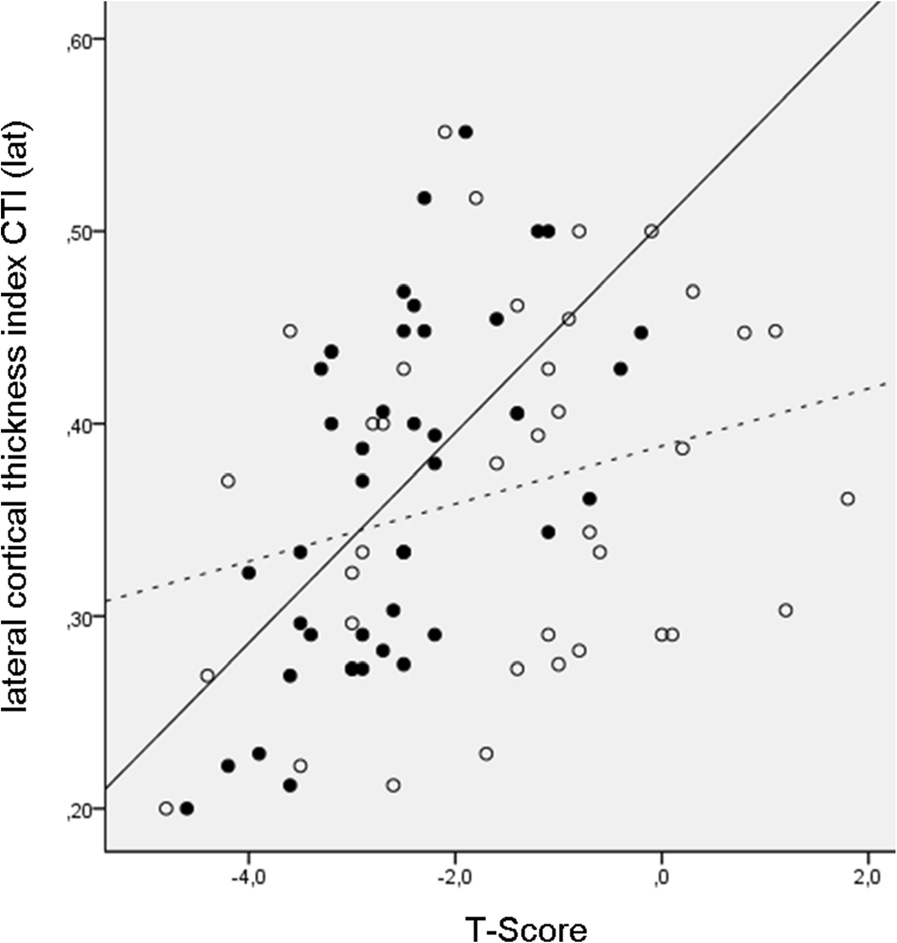
Relationship between lateral cortical thickness index CTI (lat) and T-Scores of the the total hip (black dots, black line) and lumbar spine (white dots, dashed line)

The radiographic parameters CTI (lat) and (ap) exhibited a weak negative correlation with age (*p* < 0.001, *r* = −0.336 and *p* = 0.001, *r* = −0.316), whereas no correlation was found between age and T-scores. A correlation of moderate degree was found for CTI (lat) and the T-Score at the total hip (*p* < 0.001, *r* = 0.589) and femoral neck (*p* = 0.005, *r* = 0.405) whereas the correlation at the lumbar spine was weaker (*p* = 0.002, *r* = 0.299) (Fig. 2). Similar results were seen between CTI (ap) and T-Score at the total hip (*p* < 0.001, *r* = 0.495), the femoral neck (*p* = 0.004, *r* = 0.408) and the lumbar spine (*p* = 0.010, *r* = 0.252) (Table 1).

**Table 1:** Correlation of radiographic indices with T-Scores at different locatios

		T-Score (total hip)	T-Score (femoral neck)	T-Score (spine)
Cortical index (ap)	correlation (rho r)	0.495	0.408	0.252
	significance (p)	<0.001	0.004	0.010
Cortical index (lat)	correlation (rho r)	0.589	0.405	0.299
	significance (p)	<0.001	0.005	0.002
Calcar-to-Canal ratio	correlation (rho r)	−0,292	−0.095	−0.075
	significance (p)	0.117	0.631	0.590

The lateral cortical index was further analysed in relation to BMD. At various cut-off levels it was checked for sensitivity and specificity. CTI (lat) in our study cohort showed a sensitivity of 79 % at a cut-off level of 0.4 and a specificity of 56 % in predicting a T-score ≤ −2.5 at the (total) hip (Table 2). For CTI (ap) sensitivity was even lower at comparable specificity values. A CTI (lat) ≤ 0.4 was exhibited in 57 % of patients.

**Table 2:** Sensitivity and specificity of the cortical index (lat) at different cut-off levels

	CTI (lat)	Sensitivity	Specificity
T-Score (total hip)	0.44	89 %	43 %
	0.42	82 %	48 %
	0.40	79 %	56 %
	0.38	71 %	70 %
	0.36	64 %	78 %
T-Score (femoral neck)	0.44	76 %	36 %
	0.42	74 %	43 %
	0.40	71 %	50 %
	0.38	59 %	57 %
	0.36	50 %	57 %
T-Score (spine)	0.44	91 %	39 %
	0.42	84 %	43 %
	0.40	81 %	53 %
	0.38	75 %	60 %
	0.36	69 %	63 %

Comparison between men and women revealed no differences with regard to T-Scores, radiographic indices and 25 (OH)D levels. As expected, age of female patients was significantly higher (Table 3). In female patients linear correlation of CTI (ap) and CTI (lat) with T-Scores was similar to overall results, but in men no linear correlations between radiographic indices and T-Scores could be identified. Nevertheless, in male patients the CTI (lat) predicted a T-Score ≤ −2.5 at the lumbar spine (*n* = 19) with a sensitivity of 100 % and a specificity of 73 % at a cut-off level of 0.4 (*p* = 0.018).

**Table 3:** Characteristics of female and male patients

	Female	Male	*p*
Age	80 (74–87)	75 (68–83)	0.012
DXA lumbar spine	−1.6 (−2.8 to −0.7)	−1.4 (−2.3 to −1.4)	0.352
DXA total hip	−2.6 (−3.2 to −2.2)	- 1.9 (−2.9 to −0.5)	0.073
DXA femoral neck	−3.2 (−3.9 to −2.5)	−2.6 (−3.5 to −1.4)	0.123
serum 25OH-vitamin D	8.4 (5.5 – 14,1)	9.3 (6.7 - 21.4)	0.171

The median serum 25(OH)D level was 8.7 (IQR 5.6-15.9) ng/ml. 58 % of patients presented with vitamin D levels below 10 ng/ml defined as severe deficiency. Spearman correlation was performed for radiographic parameters and serum 25(OH)D and total serum calcium. A significant, but weak correlation was indicated between CTI (lat) and 25(OH)D (*p* = 0.002, *r* = 0.293) as well as CTI (ap) and 25(OH)D (*p* = 0.003, *r* = 0.278) in the total study cohort. Using the CTI (lat) at cut-off level ≤ 0.4 predicted a severe vitamin D deficiency (10 < ng/ml) with a sensitivity of 75 % and a specificity of 66 % in the study population (*p* = 0.001). No correlation was seen between total serum calcium levels and radiographic parameters.

## Discussion

Conventional plain radiographs of the hip facilitate not only diagnosis of localized processes like osteoarthritis or fractures, but also provide the chance to detect systemic changes like osteoporosis [13, 16, 17]. Besides showing correlation with BMD, there is a possible scientific basis for using the cortical thickness as a measurement [22]. Bousson et al. [23] found that specific cortical BMD as measured with QCT showed the strongest correlation with femoral failure load. Yang et al. [24] presented data that compare the influence of area BMD (DXA), volumetric BMD (QCT) and site specific BMD in hip fractures. Their conclusion was that volumetric BMD of cortical bone as well as apparent cortical thickness provided additional information about fracture risk, as compared to area BMD alone. These results are underlined by biomechanical cadaver model testing of the femoral neck that showed the relative contribution of the cortical bone to be superior to that of trabecular bone [25].

Sah et al. [17] studied the cortical index in relation to BMD in a group of 32 patients with osteoarthritis planned for arthroplasty. In 13 patients (25 %) osteoporosis was diagnosed with a T-Score equal or below −2.5. These patients also had a significant lower CTI (ap) and CTI (lat). A significant positive correlation was demonstrated for the T-Score at the femoral neck and CTI (ap) and CTI (lat) reflected by Spearman *r* = 0.478 (*p* = 0.003) and *r* = 0.459 (*p* = 0.004), respectively. Further analysis revealed that a CTI (lat) ≤ 0.4 identifies a T-score ≤ −2.5 and subsequently osteoporosis with a sensitivity of 85 % and specificity of 79 % [17]. Our study also revealed a significant correlation between T-Scores and CTI (lat) or CTI (ap). Very similar Spearman rho values were found for T-Scores at the total hip and femoral neck. Using a cut-off level of 0.4 with the CTI (lat) to detect osteoporosis (T-Score ≤ −2.5), sensitivity was almost equal to our results (79 % at the total hip and 81 % at the spine), but specificity was considerably lower (56 % and 53 %, respectively) compared to the specificity of 79 % found by Sah et al. [17]. This means that the CTI (lat) of less than 0.4 identifies up to 81 % of patients with a T-Score ≤ −2.5. However, 44 % of patients with a T-Score > −2.5 will wrongly be diagnosed with osteoporosis according to DXA measurements. The difference of specificity between the results of Sah et al. [17] and ours are probably attributable to different patient characteristics. Patients in our study were much older (79 years vs. 67 years) and had a femoral neck fracture after low-energy trauma, and thus a high chance of established osteoporosis compared to the patients with osteoarthritis scheduled for elective THA. A CTI (lat) at a 0.4 cut-off level potentially would have a higher specificity if applied to a more generic or younger population rather than on patients in which osteoporosis must be assumed. This is supported by our results demonstrating an inverse linear correlation (*r* = −0.336, *p* < 0.001) of age with CTI (lat), but not with T-Scores. Additionally, this hypothesis is underlined by our gender dependent analysis demonstrating different results for men which were significantly younger than women. A considerably higher sensitivity (100 %) and specificity (73 %) of CTI (lat) at a cut-off level of 0.4 in predicting a T-Score ≤ −2.5 at the lumbar spine in men was demonstrated, although no linear correlation could be found. The CCR did not correlate significantly with the T-Score, equivalent to the finding of Sah et al. [17]. They attributed this result to the effect that the CCR does not compensate for differences in patient femoral length and a fixed starting point 10 cm below the mid-lesser trochanter potentially reflecting different portions of the femur depending on patient height. In opposition, measurements of cortical width in relation to overall width are normalized and consequently independent of patient height [17].

Besides BMD, 25(OH)D seems to be a major contributing factor in bone fragility. Seitz et al. [18] hypothesized that impaired bone mineralization accompanied by low serum 25(OH)D levels are a major determinant in the etiology of femoral neck fractures. Moreover, serum 25(OH)D levels are directly associated with cortical volume and width as shown by histomorphometric analysis [26] and QCT [27]. A positive linear correlation between 25(OH)D levels and bone architecture indices was also seen in our study population. Using the CTI (lat) at cut-off level ≤ 0.4 predicted a severe vitamin D deficiency (10 < ng/ml) with a sensitivity of 70 % and a specificity of 66 % in the study population (*p* = 0.001). Severe vitamin D deficiency not only is associated with impaired bone quality, but also seems to be a risk factor for adverse outcome after surgical procedures [28]. Subsequently, predefining patients warranting further expensive biochemical diagnostics by radiographic indices might effectively increase patient safety.

Apart from radiologic diagnostics, a mechanical device was lately introduced to estimate bone quality in-vivo. This torque-testing device was developed for intraoperative measurement of bone strength [10]. It showed good a correlation between the T-Score and peak torque (*r* = 0.64, *p* < 0.001) in the femoral neck of cadaveric femurs [11]. Compared to CTI (lat) measurements in the study of Sah et al. [17] and ours, estimation of the T-Score seems to be slightly more accurate, but at the price of obtaining this information later, that is intraoperatively. With regard to hip surgery, inherent disadvantages could be loss of time, necessity to switch to another implant or instruments and possibly repositioning of the patient on the operation table. Additionally, clinical experience with this device is still limited [12]. Radiographic parameters may be an alternative proxy to estimate the BMD of the proximal femur and subsequently might offer support to the orthopaedic surgeon in deciding already preoperatively whether to use cement augmentation or what type of implant to choose. Furthermore, radiographic parameters could be used as a pre-screening tool for patients warranting a more specific diagnostic work-up for osteoporosis. But it must be recognized that considerable external rotation is seen in some patients with a femoral neck fracture. Since the cross section of the femur diaphysis is not an exact circle and its cortical thickness varies, preoperative radiographic measurements of hip x-rays cannot be established in all cases unless positioning in terms of rotation is not standardized.

This study has several strengths. Apart from a higher number of included patients compared to previous studies [16, 17], a gender specific analysis was performed. Selection of elderly patients with a femoral neck fracture after low-energy trauma offered the chance to validate radiographic parameters and indices on patients in which osteoporosis must be assumed independent of DXA and T-Scores. In this study cohort a CTI (lat) ≤ 0.4 was found in 57 % which is comparable to T-Score levels ≤ −2.5 at the total hip (53 %) and femoral neck (71 %) indicating that other factors besides bone density and radiographic architecture might be involved in bone quality. Selection of patients of course is subject to bias, a major limitation on the other hand. This potentially influenced specificity of a CTI (lat) at a cut-off level of 0.4 which was substantially lower compared to the results of Sah and co-workers [17]. Another major limitation is the fact that a standardized positioning protocol for the lower extremity in obtaining preoperative hip x-rays was not used in all patients warranting consultation of postoperative x-rays in 38 %.

In summary, radiographic indices as the CTI (lat) exhibit a direct correlation to BMD and serum 25(OH)D levels. It is readily available and simple to measure at no additional costs and offers the chance for orthopaedic surgeons to estimate bone quality in terms of BMD in the emergent setting of hip fractures and thus aid in choice of the optimal implant. By using this index as a pre-screening tool patients at risk of osteoporosis or severe vitamin D deficiency can be identified and thus additionally increase patient safety. A CTI (lat) cut-off level of 0.4 is recommended for identifying patients at risk of significantly reduced BMD expressed by T-Scores ≤ −2.5 or severe vitamin D deficiency.

The study has been performed in accordance with the ethical standards as laid down in the 1964 Declaration of Helsinki and its later amendments. For this type of study formal consent is not required (retrospective study).

## Abbreviations

DXA: Dual-energy X-ray absorptiometry
BMD: Bone mineral density
CTI (ap): Cortical thickness index anteroposterior view
CTI (lat): Cortical Thickness Index lateral (axial) view
QCT: Quantitative computer tomography
QUS: Quantitative ultrasound
25(OH)D: 25-hydroxy vitamin D
